# Mitochondrial TSPO Promotes Hepatocellular Carcinoma Progression through Ferroptosis Inhibition and Immune Evasion

**DOI:** 10.1002/advs.202206669

**Published:** 2023-03-30

**Authors:** Deguo Zhang, Da Man, Jiahua Lu, Yifan Jiang, Bo Ding, Rong Su, Rongliang Tong, Junru Chen, Beng Yang, Shusen Zheng, Diyu Chen, Jian Wu

**Affiliations:** ^1^ Division of Hepatobiliary and Pancreatic Surgery Department of Surgery The First Affiliated Hospital Zhejiang University School of Medicine Hangzhou Zhejiang Province 310003 China; ^2^ NHC Key Laboratory of Combined Multi‐organ Transplantation Hangzhou Zhejiang Province 310003 China; ^3^ Key Laboratory of the Diagnosis and Treatment of Organ Transplantation Research Unit of Collaborative Diagnosis and Treatment For Hepatobiliary and Pancreatic Cancer Chinese Academy of Medical Sciences (2019RU019) Hangzhou Zhejiang Province 310003 China; ^4^ Key Laboratory of Organ Transplantation Research Center for Diagnosis and Treatment of Hepatobiliary Diseases Hangzhou Zhejiang Province 310003 China

**Keywords:** ferroptosis, hepatocellular carcinoma, immunotherapy, mitochondria, translocator protein

## Abstract

Hepatocellular carcinoma (HCC) is one of the most common malignancies with poor prognosis, and novel treatment strategies are urgently needed. Mitochondria are key regulators of cellular homeostasis and potential targets for tumor therapy. Here, the role of mitochondrial translocator protein (TSPO) in the regulation of ferroptosis and antitumor immunity is investigated and the potential therapeutic implications for HCC are assessed. TSPO is highly expressed in HCC and associated with poor prognosis. Gain‐ and loss‐of‐function experiments present that TSPO promotes HCC cell growth, migration, and invasion in vitro and in vivo. In addition, TSPO inhibits ferroptosis in HCC cells via enhancing the Nrf2‐dependent antioxidant defense system. Mechanistically, TSPO directly interacts with P62 and interferes with autophagy, leading to the accumulation of P62. The P62 accumulation competes with KEAP1, preventing it from targeting Nrf2 for proteasomal degradation. Furthermore, TSPO promotes HCC immune escape by upregulating PD‐L1 expression through Nrf2‐mediated transcription. Notably, TSPO inhibitor PK11195 combines with anti‐PD‐1 antibody showing a synergistic anti‐tumor effect in a mouse model. Overall, the results demonstrated that mitochondrial TSPO promotes HCC progression by inhibiting ferroptosis and antitumor immunity. Targeting TSPO can be a promising new strategy for HCC treatment.

## Graphical abstract

Targeting mitochondria has been recognized as a novel strategy for cancer therapy. Mitochondrial translocator protein (TSPO) is highly expressed in hepatocellular carcinoma (HCC) and promotes HCC progression and metastasis. Mechanistically, TSPO activates the expression of antioxidant genes and PD‐L1 through the P62/KEAP1/Nrf2 pathway, thereby suppressing ferroptosis and antitumor immunity. This work provides a promising mitochondrial target for HCC therapy.

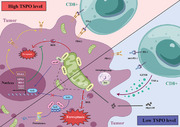

## 1 Introduction

Hepatocellular carcinoma (HCC) accounts for nearly 80% of primary liver cancers and ranks as the third most common cause of cancer‐related deaths worldwide.^[^ ^1^ ^]^ Currently, sorafenib and lenvatinib are the first‐line therapy for advanced HCC, but they provide limited benefit to patients, extending survival rates by only 3 months.^[^ ^2^, ^3^ ^]^ In recent years, immune checkpoint blockade (ICB), especially targeting the programmed death‐1 (PD‐1)/PD‐1 ligand 1 (PD‐L1) axis, has been a breakthrough strategy for the treatment of cancers.^[^ ^4^ ^]^ However, the objective response rate to PD‐1/PD‐L1 blockade monotherapy is only 15–20% in advanced HCC patients.^[^ ^5^, ^6^ ^]^ Increasing evidence reveals that the clinical response to anti‐PD‐1/PD‐L1 is highly dependent on PD‐L1 expression on tumor cells and T lymphocyte infiltration.^[^ ^7^, ^8^ ^]^ Therefore, an in‐depth understanding of the regulatory process of PD‐L1 expression may facilitate the development of novel therapeutic strategies to improve the efficacy of PD‐1/PD‐L1 blockade.

Ferroptosis is a new type of regulated cell death (RCD) that has been implicated in various physiological and pathological processes.^[^ ^9^ ^]^ The core event of ferroptosis is excessive ROS‐mediated lipid peroxidation, which eventually leads to plasma membrane damage and cell death.^[^ ^10^ ^]^ To cope with ROS‐induced oxidative damage, cells have developed an efficient antioxidant defense system, and Nrf2 is the master transcriptional regulator of antioxidant response.^[^ ^11^ ^]^ Studies have found that cancer cells are more susceptible to ferroptosis due to high load of ROS and unique metabolic characteristics.^[^ ^12^ ^]^ In addition, induction of ferroptosis could improve the efficacy of immunotherapy and enhance antitumor immune response.^[^ ^13^, ^14^ ^]^ Therefore, triggering ferroptosis become a new therapeutic strategy for cancer therapy.

Mitochondria, a double‐membrane‐bound organelle, are the energy factories of cells.^[^ ^15^ ^]^ As the main site of ROS generation and iron metabolism, mitochondria play a central role in the induction of ferroptosis.^[^ ^16^ ^]^ The 18 kDa translocator protein (TSPO) is a highly conserved transmembrane protein primarily localized at the outer mitochondrial membrane.^[^ ^17^, ^18^ ^]^ TSPO is widely expressed throughout the body and is involved in a broad range of mitochondrial functions, including oxidative stress regulation, iron homeostasis and cholesterol transport.^[^ ^19^, ^20^, ^21^, ^22^, ^23^ ^]^ It has been reported that TSPO expression is increased in a variety of cancer types and is associated with tumor progression and poor prognosis.^[^ ^24^, ^25^, ^26^, ^27^, ^28^ ^]^ Additionally, bioinformatics analysis indicated that TSPO expression was associated with immune cell infiltration.^[^ ^29^ ^]^ However, the role of TSPO in the regulation of ferroptosis and antitumor immunity during tumorigenesis is unclear.

In this study, we investigated the role and underlying mechanism of TSPO in promoting HCC progression by regulating ferroptosis and antitumor immunity. Furthermore, we evaluated the efficacy and safety of combination therapy with TSPO inhibitor PK11195 and anti‐PD‐1 antibody in a mouse model. Our results indicate that TSPO may be an important new target for HCC therapy.

## 2 Results

### 2.1 TSPO Is Significantly Upregulated in HCC and Indicates Poor Prognosis

**Figure 1:**
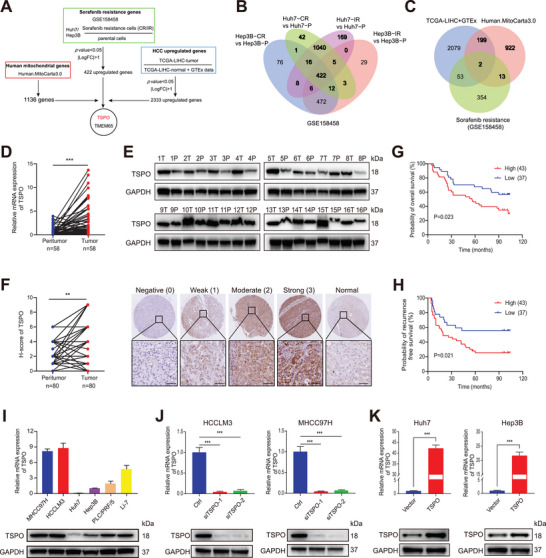
TSPO is upregulated and associated with a poor prognosis in HCC. A) TSPO screening process based on the GEO, TCGA, GTEx, and MitoCarta3.0 databases. B) Sorafenib‐resistant HCC cell lines were established by increasing concentrations (IR) or continuously high concentrations (CR) of sorafenib (GSE158458). 422 up‐regulated expressed genes were identified between the four sorafenib‐resistant cell lines and their sorafenib‐responsive ancestors. C) The GEO, TCGA, GTEx and MitoCarta3.0 databases were analyzed by Venn diagram, and 2 candidate mitochondrial genes (TSPO and TMEM65) were finally confirmed. D) TSPO mRNA expression in paired HCC tissues and adjacent non‐tumor tissues evaluated by qRT‐PCR (*n* = 58). E) TSPO protein expression in paired HCC tissues and adjacent non‐tumor tissues evaluated by Western blot (*n* = 16). F) H‐score and representative IHC images of TSPO from TMA in normal liver tissues and HCC tissues, which were divided into low and high groups according to the staining intensity. G,H) Kaplan–Meier survival analyses of OS and RFS in different TSPO expression groups (*n* = 80). I) Basal TSPO expression in 6 HCC cell lines using qRT‐PCR and Western blot. J,K) Validation of TSPO expression after knockdown or overexpression in the indicated cell lines using qRT‐PCR and Western blot. **p < 0.01, ***p < 0.001. The data are expressed as the mean±SD of three independent experiments. T, tumor tissues; P, para‐tumor normal tissues; OS, overall survival; RFS, recurrence free survival.

Accumulating evidences indicate that ferroptosis is an intrinsic tumor suppressor mechanism.^[^ ^30^ ^]^ However, the mechanisms by which tumor cells develop ferroptosis resistance during tumor development and progression remains unclear. Several studies have shown that ferroptosis inhibition plays an important role in the development of sorafenib resistance in HCC.^[^ ^31^, ^32^ ^]^ By analyzing the sorafenib‐resistant HCC cell models, we found that 422 genes were notably up‐regulated and may be involved in the suppression of ferroptosis (**Figure** **1**A,B). Mitochondria are the main source of cellular ROS and are closely associated with ferroptosis,^[^ ^33^ ^]^ we therefore focused on 1136 mitochondrial functional genes. Meanwhile, we screened 2333 genes up‐regulated in HCC from the TCGA and GTEx database (Figure S1A,B, Supporting Information) and finally identified 2 candidate mitochondrial genes that may inhibit ferroptosis during hepatocarcinogenesis (Figure 1A–C). Previous studies have implicated TSPO may play a role in tumor, thus we chose TSPO for further investigation.

To explore the expression of TSPO in tumors, we analyzed data from TIMER database and found that TSPO mRNA expression is increased in a variety of tumors including HCC (Figure S1C, Supporting Information). Similarly, the GEO database was used to identify the elevated expression of TSPO in HCC (Figure S1D, Supporting Information). We further validated the elevated expression of TSPO in HCC clinical specimens using quantitative real‐time PCR (qRT‐PCR) (*p* < 0.001) and Western blot (Figure 1D,E). In addition, we performed immunohistochemistry (IHC) using tissue microarrays (TMA) containing 80 pairs of HCC samples. The results showed that TSPO protein expression in HCC tissues was higher than that in adjacent non‐tumor tissues (*p* = 0.002) (Figure 1F), consistent with the HPA database analysis (Figure S1E, Supporting Information).

Survival analysis demonstrated that OS and RFS times were shorter in patients with high TSPO expression than those with low TSPO expression (Figure 1G,H). Similar results were obtained from the KM Plotter database (Figure S1F,G, Supporting Information). Next, we analyzed the relationship between TSPO expression levels and clinicopathological features in HCC cases. As a result, elevated TSPO expression was associated with poor tumor differentiation (*p* = 0.015) and advanced TNM stage (*p* = 0.027) (Table S1, Supporting Information). Importantly, multivariate Cox regression analysis revealed that high TSPO expression was an independent risk factor for both OS and RFS (hazard ratio, 1.445 and 1.410, respectively; 95% CI 1.027–2.035 and 1.020–1.948, respectively) (Table S2, Supporting Information).

We further examined the expression levels of TSPO in 6 HCC cell lines and observed high TSPO expression in HCCLM3 and MHCC97H cells, moderate expression in Li‐7 and PLC/PRF/5 cells, and low expression in Huh7 and Hep3B cells (Figure 1I). To investigate the role of TSPO in HCC, TSPO knockdown (HCCLM3 and MHCC97H), and overexpression (Huh7 and Hep3B) cell lines were respectively constructed (Figure 1J,K). Collectively, TSPO is highly expressed in HCC with poor prognosis and is a potential biomarker for diagnosis and prognosis.

### 2.2 TSPO Promotes HCC Cell Proliferation, Invasion, and Metastasis In Vitro and In Vivo

**Figure 2:**
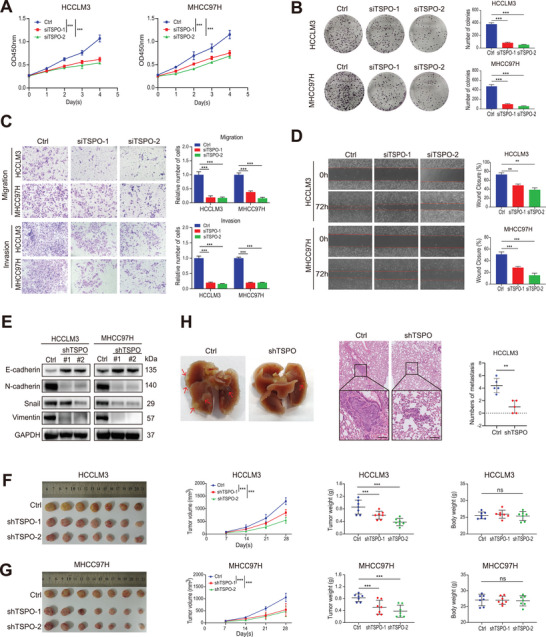
Knockdown of TSPO inhibits HCC cell proliferation, invasion and metastasis in vitro and in vivo. A,B) The proliferation of HCCLM3 and MHCC97H cells was detected by CCK‐8 and colony formation assays. C,D) The migratory and invasive capabilities of HCCLM3 and MHCC97H cells were evaluated using wound healing and transwell assays. E) The protein levels of EMT markers in HCCLM3 and MHCC97H cells were detected by Western blot. F,G) Representative images of xenograft tumors in nude mice and statistical analyses of tumor volumes, tumor weights, and body weights in the different groups (*n* = 7). H) Representative images and quantification of metastatic lung nodules in nude mice (red arrows marked, *n* = 5). **p < 0.01, ***p < 0.001. n.s, not significant. The data are expressed as the mean±SD of three independent experiments.

In the CCK‐8 and colony formation assays, TSPO knockdown inhibited proliferation and colony formation of HCCLM3 and MHCC97H cells (**Figure** **2**A,B). Wound healing and transwell assays demonstrated that silence of TSPO induced the suppression of the metastasis ability in HCC cells (Figure 2C,D). The overexpression of TSPO had the opposite effects on cell proliferation (Figure S2A,B, Supporting Information) and metastasis (Figure S2C,D, Supporting Information). Since epithelial‐mesenchymal transition (EMT) contributes to enhanced migration and metastasis of tumor cells, we examined whether the EMT process could be mediated by TSPO. The results showed that TSPO expression was negatively correlated with E‐cadherin, but positively correlated with N‐cadherin, snail and vimentin (Figure 2E and Figure S2E, Supporting Information). To further investigate the role of TSPO on tumor growth and metastatic potential in vivo, we established subcutaneous xenograft tumor and lung metastasis models in nude mice. Consistent with the in vitro result, following the knockdown of TSPO, the reduction of tumor burden and lung metastasis were observed (Figure 2F–H), whereas overexpression of TSPO resulted in the opposite outcome (Figure S2F–H, Supporting Information). Taken together, our results demonstrate that TSPO facilitates HCC tumorigenesis and metastasis both in vitro and in vivo.

### 2.3 TSPO Inhibits Ferroptosis in HCC Cells

**Figure 3:**
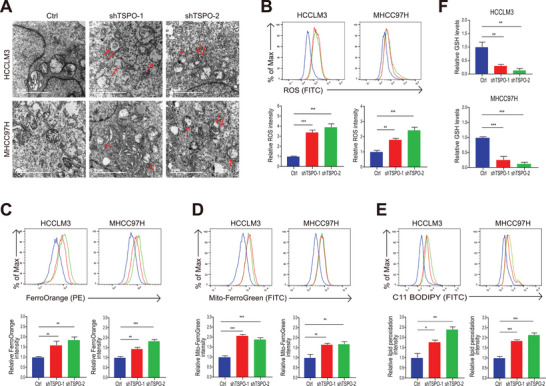
TSPO inhibits ferroptosis in HCC cells. A) The morphology of mitochondria in HCCLM3 and MHCC97H cells was observed by TEM. Single arrows indicate damaged mitochondria and double arrows indicate autophagosomes. B) The intracellular ROS of HCCLM3 and MHCC97H cells was detected by flow cytometry using DCFH‐DA. C,D) The intracellular and intramitochondrial Fe^2+^ of HCCLM3 and MHCC97H cells were detected by flow cytometry using FerroOrange and Mito‐FerroGreen, respectively. E) The accumulation of lipid ROS of HCCLM3 and MHCC97H cells was detected by flow cytometry using C11‐BODIPY 581/591. F) The intracellular GSH of HCCLM3 and MHCC97H cells was detected using a GSSG/GSH quantification kit. *p < 0.05, **p < 0.01, ***p < 0.001. The data are expressed as the mean±SD of three independent experiments. TEM, transmission electron microscopy. DCFH‐DA, 20,70‐dichlorodihydrofluorescein diacetate.

To confirm whether TSPO localizes to the mitochondria, we performed immunofluorescence colocalization staining using the mitochondria‐specific dye MitoTracker and found that TSPO was highly colocalized with MitoTracker (Figure S3A,B, Supporting Information). The mitochondrial localization of TSPO was further confirmed using mitochondrial isolation method (Figure S3C, Supporting Information). As an important mitochondrial protein, TSPO has been reported to be involved in cholesterol transport from intracellular to mitochondria.^[^ ^34^ ^]^ Similarly, cholesterol accumulation was observed in tumor cells after down‐regulation of TSPO (Figure S3D, Supporting Information). To investigate the putative role of TSPO in the ferroptosis, we examined the characteristic indicators associated with ferroptosis. Since changes in mitochondrial morphology are the main feature of ferroptosis, transmission electron microscopy was performed to study mitochondrial morphology. The results showed that mitochondria in TSPO‐silenced cells were smaller and had fewer cristae (**Figure** **3**A). Interestingly, we found that TSPO knockdown resulted in an increased number of autophagosomes. Furthermore, biochemical characterization results indicated that TSPO inhibition increased the intracellular ROS (Figure 3B), the level of intracellular and intramitochondrial Fe^2+^ (Figure 3C,D) and the accumulation of lipid ROS (Figure 3E) while decreasing intracellular GSH (Figure 3F). These results indicate that TSPO knockdown induces mitochondrial damage and promotes ferroptosis in HCC cells.

### 2.4 TSPO Mediates Autophagy Inhibition and P62 Accumulation via Interaction with P62

**Figure 4:**
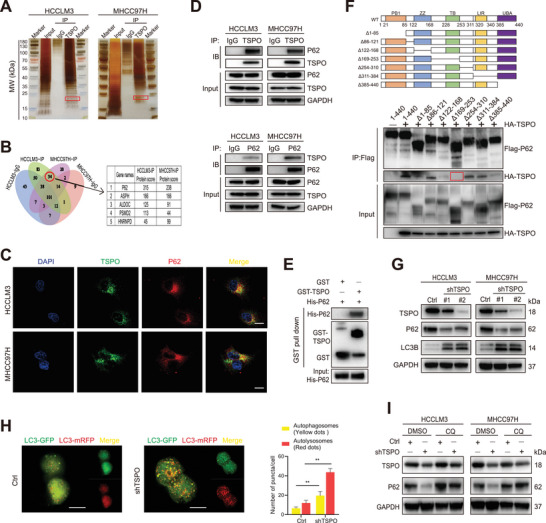
TSPO mediates autophagy inhibition and P62 accumulation via interaction with P62. A) IP and silver staining was performed in HCCLM3 and MHCC97H cells with TSPO antibody. B) The TSPO interacting proteins were identified by mass spectrometry. C) Immunofluorescence assay showed the colocalization of TSPO with P62 in HCCLM3 and MHCC97H cells (scale bar, 20 µm). D,E) Co‐IP and GST pull‐down assays to confirm the protein interaction between TSPO and P62 in vivo and in vitro, respectively. F) Co‐IP assays using Flag antibody in HEK293T cells transfected with Flag‐tagged p62 truncated mutants and HA‐TSPO. G) The protein levels of autophagy markers (p62 and LC3B) in HCCLM3 and MHCC97H cells were detected by Western blot. H) Autophagy flux was monitored by mRFP‐GFP‐LC3B adenovirus infection. Representative images show autophagosomes (yellow dots) and autolysosomes (red dots; scale bar, 20 µm). I) The addition of CQ inhibited autophagy caused by TSPO knockdown. **p < 0.01. The data are expressed as the mean±SD of three independent experiments. IP, immunoprecipitation. Co‐IP, co‐immunoprecipitation. GST, glutathione‐S‐transferase. CQ, chloroquine.

To investigate the molecular mechanisms of TSPO action in HCC cells, immunoprecipitation (IP) was performed with TSPO antibody in HCCLM3 and MHCC97H cells (**Figure** **4**A). From the Venn diagram, utilizing mass spectrometry, we screened out 34 potential candidates which could interact with TSPO in HCC cells (Figure 4B). Among these potential interacting proteins, p62 attracted our concentration as it has been confirmed to be an important substrate for autophagic process and showed the highest protein score. Database analysis showed that P62 was highly expressed in HCC and positively correlated with TSPO expression, and its overexpression was associated with poor prognosis (Figure S4A–C, Supporting Information). In addition, P62 has been shown to play an important role in the occurrence and development of liver cancer. Thus, we selected P62 as a potential TSPO interactor for further analysis. Then immunofluorescence experiment was performed to demonstrate the colocalization of TSPO and P62 in HCC cells (Figure 4C). We also carried out the Co‐IP and GST pull‐down assays, and the results showed that p62 could directly interact with TSPO in vivo and in vitro, respectively (Figure 4D,E). To further discover the TSPO interactive regions that existed in p62, we constructed 7 kinds of p62 deletion mutation vectors with FLAG tag. The results showed that TSPO‐HA failed to co‐precipitate with P62‐Flag containing a deletion of amino acids 169–253 (Figure 4F).

According to these mentioned above, it was suggested that TSPO‐P62 interaction may lead to interference with the p62‐mediated autophagy. Upon TSPO knockdown, we observed increased LC3B levels and reduced p62 levels in HCC cells, indicating enhanced autophagosome synthesis (Figure 4G). This result was also confirmed by transmission electron microscopy (Figure 3A). To further monitor the autophagic flux, HCC cells were infected with a GFP‐mRFP‐LC3 adenovirus. Our data showed that knockdown of TSPO significantly increased the number of autophagosomes and autolysosomes, suggesting activation of the autophagic process (Figure 4H). Moreover, the addition of CQ (a late‐stage autophagy inhibitor) attenuated the degradation of P62 caused by TSPO knockdown (Figure 4I). Together, these results suggest that TSPO mediates autophagy inhibition and P62 accumulation via interaction with P62.

### 2.5 TSPO Inhibits Ferroptosis through P62/KEAP1/Nrf2 Antioxidant Pathway

**Figure 5:**
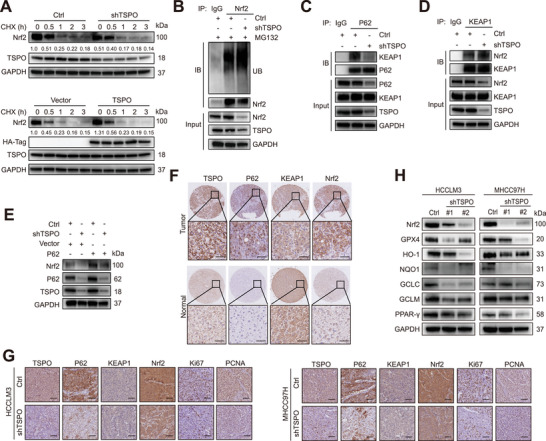
TSPO inhibits ferroptosis through P62/KEAP1/Nrf2 antioxidant pathway. A) HCCLM3 cells with stable TSPO knockdown and overexpression were treated with 100 µg mL^−1^ cycloheximide (CHX) for the indicated times, then lysed and subjected to immunoblotting. B) Cells were transfected with HA‐Ub plasmid and treated with MG132 (10 µm) for 10 h. The ubiquitination levels of Nrf2 were detected by western blot analysis. C) Co‐IP assays were performed to detect the interaction between P62 and KEAP1 when TSPO was knockdown. D) Co‐IP assays were performed to detect the interaction between KEAP1 and Nrf2 when TSPO was knockdown. E) P62 was overexpressed in TSPO knockdown cells, and then Nrf2 expression was examined by Western blot. F,G) Representative images of IHC staining of TSPO, P62, KEAP1, and Nrf2 in TMA samples and subcutaneous xenografts tumor tissues. H) The protein levels of downstream antioxidant genes regulated by Nrf2 in HCCLM3 and MHCC97H cells were detected by Western blot.

Previous studies have demonstrated that ferroptosis is an autophagy‐dependent cell death, and P62 is involved in the regulation of ferroptosis by activating Nrf2.^[^ ^35^, ^36^ ^]^ Under basal conditions, Nrf2 is repressed by the E3 ubiquitin ligase KEAP1, which facilitates the ubiquitination and proteasomal degradation of Nrf2. Excessive accumulation of P62 can compete with Nrf2 for binding to KEAP1, resulting in Nrf2 stabilization. Immunofluorescence and Co‐IP assays were performed to verify the colocalization and interaction between P62, KEAP1, and Nrf2 (Figure S5A–E, Supporting Information). Therefore, we hypothesized that high expression of TSPO in HCC mediated P62 accumulation thereby disrupting KEAP1‐Nrf2 association, leading to Nrf2 stabilization, and ultimately protecting cells against ferroptosis. To determine whether TSPO affects Nrf2 protein stability, HCCLM3 cells were treated with cycloheximide (CHX) after TSPO knockdown or overexpression. The results showed that TSPO knockdown promoted Nrf2 degradation and TSPO overexpression exerts an opposite role (**Figure** **5**A). The ubiquitination assay demonstrated that TSPO inhibition could promote the ubiquitination level of Nrf2 (Figure 5B). Co‐IP assays showed that TSPO silencing inhibited the interaction of P62 with KEAP1, whereas it enhanced the interaction of KEAP1 with Nrf2 (Figure 5C,D). Importantly, P62 overexpression significantly attenuated Nrf2 degradation induced by TSPO inhibition (Figure 5E). IHC analysis of TMA and subcutaneous xenografts tumor tissues showed that TSPO was positively correlated with P62 and Nrf2 (Figure 5F,G). As Nrf2 can activate the transcription of plenty of antioxidant genes, we examined their expression levels and found that they were significantly decreased after TSPO knockdown (Figure 5H and Figure S5F, Supporting Information). Collectively, these results indicate that TSPO inhibits ferroptosis through P62/KEAP1/Nrf2 antioxidant pathway.

### 2.6 Nrf2 Overexpression Reverses Ferroptosis and Phenotype Changes Induced by TSPO Knockdown

To further confirm that TSPO inhibited ferroptosis and induced oncogenic phenotype in HCC cells through Nrf2, we performed rescue experiments by overexpressing Nrf2. As expected, overexpression of Nrf2 alleviated the mitochondrial injury induced by TSPO inhibition (Figure S6A, Supporting Information). Similar results have been observed in the intracellular ROS (Figure S6B, Supporting Information), the level of intracellular and intramitochondrial Fe^2+^ (Figure S6C,D, Supporting Information), the accumulation of lipid ROS (Figure S6E, Supporting Information), and intracellular GSH (Figure S6F, Supporting Information). Furthermore, overexpression of Nrf2 abolished the inhibitory effect of TSPO knockdown on cell proliferation (Figure S6G,H, Supporting Information). These data suggest that Nrf2 overexpression rescues ferroptosis and phenotypic changes induced by TSPO knockdown.

### 2.7 TSPO Promotes Tumor Immune Escape by Upregulating PD‐L1 Expression through Nrf2‐Mediated Transcription

**Figure 6:**
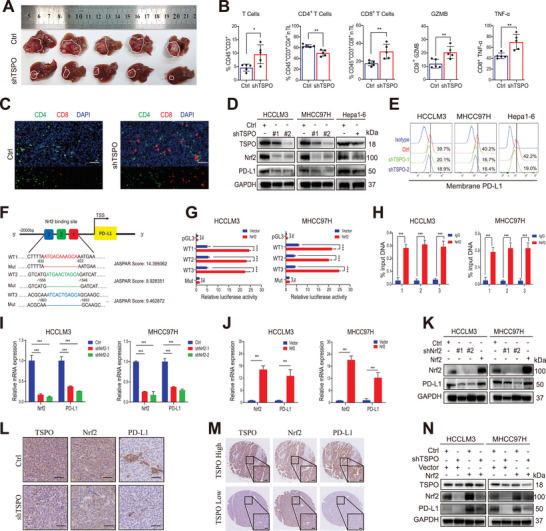
TSPO promotes tumor immune escape by upregulating PD‐L1 expression through Nrf2‐mediated transcription. A,B) Representative images of orthotopic liver cancer in C57/BL6 mice and statistical analyses of tumor infiltrated lymphocytes (total T cells, CD4^+^T cells, CD8^+^T cells, and functional state of CD8^+^T cells) by flow cytometric (*n* = 5). C) Immunofluorescence staining of tissue sections showed the tumor infiltrated lymphocytes (CD4^+^T cells and CD8^+^T cells; scale bar, 50 µm). D,E) The protein levels of PD‐L1 in HCCLM3, MHCC97H, and Hepa1‐6 cells were detected by Western blot and flow cytometric. F) The predicted binding site of Nrf2 in the PD‐L1 promoter region according to the Jaspar database. G,H) Dual luciferase reporter and ChIP assays were performed to analyze the direct interaction between Nrf2 and PD‐L1. I–K) The mRNA and protein expression of PD‐L1 was regulated by Nrf2 knockdown or overexpression. L,M) Representative images of IHC staining with TSPO, Nrf2, and PD‐L1 antibodies in orthotopic liver cancer tissues and TMA. N) Western blot analysis demonstrated that the reduction in PD‐L1 expression induced by TSPO knockdown was abolished by Nrf2 overexpression. *p < 0.05, **p < 0.01, ***p < 0.001. The data are expressed as the mean±SD of three independent experiments. TIL, tumor‐infiltrating lymphocyte. ChIP, chromatin immunoprecipitation.

Recent studies have suggested that ferroptosis plays an important role in antitumor immunity, especially in T cell‐mediated immune response.^[^ ^13^, ^37^ ^]^ Thus, to further explore the effects and underlying mechanisms of ferroptosis on antitumor immunity, the orthotopic HCC model was established using Hepa1‐6 cells in C57/BL6 mice. The results showed that TSPO knockdown significantly suppressed tumor growth and facilitated T‐cell infiltration (**Figure** **6**A). Among them, the proportion and absolute number of CD8^+^ T cells increased, and the proportion of CD4^+^ T cells decreased correspondingly, but the number did not change significantly (Figure 6B and Figure S7A,B, Supporting Information). This phenomenon was further confirmed by immunofluorescence (Figure 6C). In the shTSPO Hepa1‐6 bearing mice, the increased proportion of effector CD8^+^ T cells with cytotoxic activity (Granzyme B^+^ and TNF‐*α* ^+^) was observed (Figure 6B and Figure S7C, Supporting Information). During the immune escape process, PD‐1/PD‐L1 has been verified to be the necessary signal for inducing the exhaustion and dysfunction of cytotoxic T lymphocytes. To detect whether the enhanced cytotoxic activity of CD8^+^ T cell is mediated by PD‐L1 blockade, the expression levels of PD‐L1 in HCC cells were analyzed. The results showed that TSPO knockdown significantly decreased PD‐L1 mRNA and protein levels in HCC cells compared to the control group (Figure 6D and Figure S7D, Supporting Information). Similar results were obtained when analyzing the expression levels of PD‐L1 in the membrane of HCC cells using flow cytometry (Figure 6E).

Since Nrf2 is an important and ubiquitously expressed transcription factor in tumor cells, we hypothesized that PD‐L1 downregulation caused by TSPO knockdown is regulated at the transcriptional level through Nrf2. Then, we analyzed possible correlations between Nrf2 and PD‐L1 using the TIMER database and found that PD‐L1 positively correlated with Nrf2 in various tumors including liver cancer (*r* = 0.440) (Figure S7E, Supporting Information). This result was verified in HCC clinical specimens (*r* = 0.318) (Figure S7F, Supporting Information). Simultaneously, we used the Jaspar database to predict the potential Nrf2 binding sites within the promoter region of PD‐L1, and found eleven‐base motifs (Figure S7G, Supporting Information) and three potential sites (Figure 6F). Dual luciferase reporter assays (Figure 6G) and ChIP assays (Figure 6H) confirmed the direct interaction between Nrf2 and PD‐L1. Consistently, PD‐L1 mRNA and protein expression were decreased in Nrf2 knockdown HCC cells, whereas Nrf2 overexpression led to the opposite outcome (Figure 6I–K). IHC staining of orthotopic HCC tissues and TMA further confirmed that TSPO and Nrf2 were positively correlated with PD‐L1 (Figure 6L,M). Similar results were obtained by Western blot analysis of HCC clinical specimens (Figure S7H, Supporting Information). Finally, overexpression of Nrf2 effectively rescued the reduction of PDL1 protein expression caused by TSPO knockdown in HCC cells (Figure 6N). Together, these results suggest that TSPO upregulates the expression of PD‐L1 through Nrf2‐mediated transcription, thereby promoting immune evasion of HCC cells.

### 2.8 TSPO Inhibitor Promotes Ferroptosis and Improves Anti‐PD‐1 Efficacy in a Mouse Model

**Figure 7:**
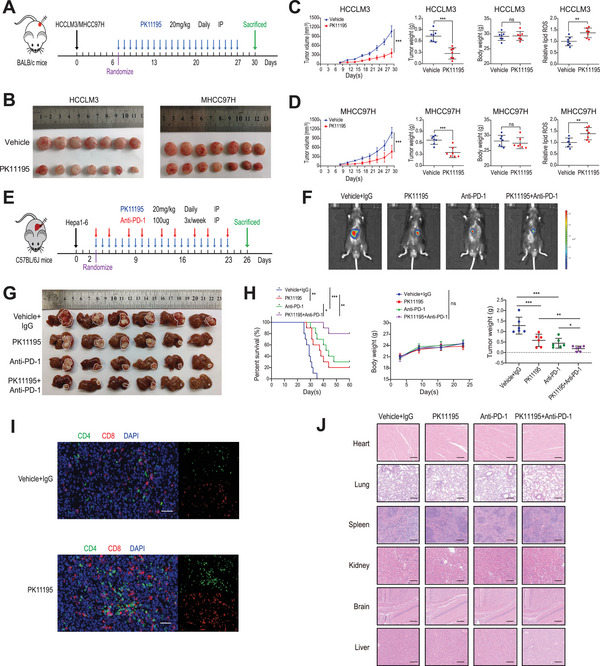
TSPO inhibitor promotes ferroptosis and improves anti‐PD‐1 efficacy in a mouse model. A) Timetable of construction of subcutaneous xenograft tumor model in nude mice and PK11195 treatment strategy. B–D) Representative images of xenograft tumors in nude mice and statistical analyses of tumor volumes, tumor weights, body weights, and lipid ROS levels (*n* = 7). E) Timetable of construction of orthotopic HCC model in C57/BL6 mice and PK11195 and anti‐PD‐1 antibody treatment strategy. F) Representative bioluminescence images of C57/BL6 mice with orthotopic tumor after 21 days of treatment (*n* = 6). G,H) Representative images of orthotopic HCC in C57/BL6 mice and statistical analyses of survival time, tumor weights, and body weights. I) Immunofluorescence staining of tissue sections showed the tumor infiltrated lymphocytes (CD4^+^T cells and CD8^+^T cells; scale bar, 50 µm). J) Representative images of HE‐stained tissue specimens (heart, lung, spleen, kidney, brain, and liver). *p < 0.05, **p < 0.01, ***p < 0.001. IP, intraperitoneal injection.

To evaluate the therapeutic potential of TSPO, we used the TSPO inhibitor PK11195 for further validation in mouse models. Subcutaneous xenograft tumor models were established in nude mice and randomized to receive vehicle control or PK11195 (20 mg kg^−1^) daily via intraperitoneal injection (**Figure** **7**A). The results showed that PK11195 effectively inhibited tumors size and weight and increased lipid ROS levels compared with vehicle control (Figure 7B–D). The IHC staining results showed that PK11195 inhibited the expression of TSPO and downstream P62‐KEAP1‐Nrf2 signal axis (Figure S8A,B, Supporting Information). These results indicate that PK11195 induces ferroptosis in vivo by targeting TSPO.

**Figure 8:**
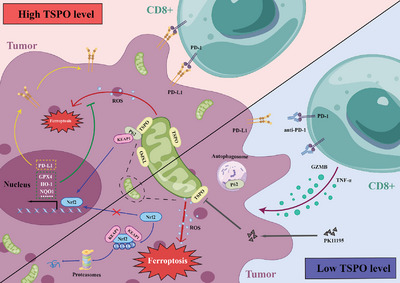
A model illustrating the role of TSPO in regulating ferroptosis and antitumor immunity in HCC cells. TSPO interacts with p62 and stabilizes Nrf2 protein in HCC cells, which promotes the expression of antioxidant genes and PD‐L1, leading to ferroptosis inhibition and immune escape (up). However, inhibition of TSPO by PK11195 promotes the ubiquitination and proteasomal degradation of Nrf2, thereby promoting ferroptosis, sensitizing to anti‐PD‐1 immunotherapy and enhancing CD8^+^T cells‐mediated cell killing (down).

Recent studies revealed that ferroptosis inducers enhanced the efficacy of anti‐PD‐1/PD‐L1 therapy.^[^ ^13^, ^38^ ^]^ To examine the potential synergistic effect, orthotopic HCC models were constructed in C57/BL6 mice and randomized to receive single or combination therapy with PK11195 (20 mg kg^−1^, daily) and anti‐PD‐1 antibody (100 µg, 3 times a week) via intraperitoneal injection (Figure 7E). We observed that the monotherapy significantly inhibited tumor growth and improved the overall survival, but combination therapy produced better anticancer therapeutic effects (Figure 7F–H). Immunofluorescence results showed that PK11195 treatment effectively increased the infiltration of CD8^+^ T cells (Figure 7I). Additionally, combination therapy did not exert an effect on body weight or the indicators of heart, liver, and kidney function (Figure 7H and Figure S8C, Supporting Information). The H&E staining results also indicated no noticeable substantial damage or inflammation lesions among major organs, including heart, lung, spleen, kidney, brain and liver (Figure 7J). Hence, PK11195 seems to be a good candidate for further research as its potency and favorable safety profile. Collectively, our findings suggest that PK11195 promotes ferroptosis and improves the efficacy of anti‐PD‐1 antibody in a mouse model. Based on our data, we propose a hypothetical model as illustrated in **Figure** **8**.

## 3 Discussion

Due to the critical role of mitochondria in maintaining cellular function and survival, there is a growing interest in targeting mitochondria as a therapeutic strategy for tumors.^[^ ^39^ ^]^ Research shows that mitochondria of tumor cells are reprogrammed to provide tumor cells with the ability to survive in a hostile microenvironment, such as hypoxia and nutritional deficiencies.^[^ ^40^ ^]^ The alterations in mitochondrial structure and function of tumor cells are the basis of targeting mitochondria therapy. Currently, although there are clinical trials of inhibitors targeting mitochondrial metabolic enzymes,^[^ ^41^, ^42^ ^]^ these inhibitors still face challenges such as including insufficient tumor targeting and low mitochondria permeability. The mitochondrial TSPO is located on the outer mitochondrial membrane and its expression is increased in various tumors compared with normal tissues, suggesting that TSPO is a promising tumor‐specific target (Figure S1C, Supporting Information). A previous study showed that high TSPO expression was observed in HCC by IHC staining.^[^ ^43^ ^]^ However, the function role and molecular mechanism of TSPO in HCC is unclear. In this study, we confirmed that TSPO expression was increased in HCC and correlated with poor prognosis (Figure 1D–H and Figure S1D–G, Supporting Information). In addition, loss‐ and gain‐of‐function experiments indicated that TSPO promoted HCC cell proliferation, invasion, and metastasis in vitro and in vivo (Figure 2 and Figure S2, Supporting Information). Thus, TSPO is a candidate oncogene and a potential target for mitochondrial therapy in HCC.

Elevated levels of ROS are an important common feature of cancer and can promote cancer progression. When ROS levels exceed the endogenous antioxidant buffering capacity, excessive production of ROS can induce cell damage or even death.^[^ ^44^ ^]^ Ferroptosis is a ROS‐dependent RCD and has attracted great attention as it may offer new opportunities for cancer therapy.^[^ ^45^ ^]^ Although mitochondria are strongly related to ferroptosis, the role and mechanism of mitochondria in tumor progression by regulating ferroptosis remain largely elusive. Here, we found that TSPO inhibited ferroptosis in HCC cells through Nrf2‐mediated upregulation of antioxidant gene expression, thereby promoting HCC development (Figures 3 and 5H, and Figure S5F, Supporting Information). Rescue experiments further confirmed that TSPO regulated the malignant phenotype and ferroptosis of HCC cells dependent on the stable expression of Nrf2 (Figure S6, Supporting Information). Mitochondrial dysfunction is one of the characteristics of ferroptosis. For example, erastin, a classic ferroptosis inducer, can promote ferroptosis by inducing mitochondrial dysfunction.^[^ ^10^ ^]^ In our study, silencing of TSPO expression also resulted in mitochondrial damage and dysfunction. Additionally, studies have reported that upregulation of Nrf2 expression in tumor cells could protect mitochondrial function,^[^ ^46^ ^]^ however, the regulatory mechanism of Nrf2 has not been fully demonstrated. Our results suggested that TSPO interacted with autophagic receptor P62, leading to autophagy inhibition and P62 accumulation (Figure 4). The stabilization of Nrf2 depended on the competitive binding of P62 to KEAP1, which mediated ubiquitination and proteasome degradation of Nrf2 (Figure 5A–E and Figure S5A–E, Supporting Information). This result is consistent with findings reported previously for the P62‐KEAP1‐Nrf2 pathway.^[^ ^47^ ^]^ In addition, we performed immunohistochemical analysis of clinical specimens to further determine the positively correlated expression of TSPO/P62/KEAP1/Nrf2 in HCC (Figure 5F,G). Collectively, our study shows that upregulated TSPO in HCC cells suppresses ferroptosis by maintaining intracellular redox homeostasis, thereby promoting HCC progression.

Recent research has found that ferroptosis induction could augment antitumor immune responses, however, the underlying mechanism is not well understood. A study reported that TYRO3, a member of receptor tyrosine kinase, inhibited ferroptosis and reduced the antitumor effects of cytotoxic T cells.^[^ ^13^ ^]^ In the present study, TSPO inhibition promoted ferroptosis and increased CD8^+^ T cells infiltration and cytotoxicity (Figure 6A–C and Figure S7A–C, Supporting Information). Mechanistically, Nrf2 could bind to multiple sites within the promoter region of PD‐L1 and promote transcriptional expression of PD‐L1 (Figure 6D–K and Figure S7D–G, Supporting Information). While we conducted the study, Zhu et al. reported that Nrf2 induced by ROS significantly increased PD‐L1 mRNA expression in colorectal cancer cell, which strongly supported our observations.^[^ ^48^ ^]^ Importantly, we further validated the regulation of PD‐L1 expression by Nrf2 through clinical specimens and animal models (Figure 6L,M and Figure S7H, Supporting Information). Thus, our study revealed an underlying mechanism by which ferroptosis of tumor cells regulates antitumor immune response. Furthermore, our data provide a new perspective on the involvement of tumor cell mitochondria in immune evasion, which may help develop new therapeutic strategies. These findings reveal an unprecedented relationship between mitochondria, ferroptosis, and antitumor immunity and highlight the critical role of TSPO in suppressing ferroptosis and promoting immune escape during HCC progression. However, further studies are required to elucidate the detailed underlying mechanisms.

Furthermore, we evaluated the therapeutic effect of TSPO inhibitor PK11195 in vivo. Previous work showed that PK11195 treatment inhibited proliferation and sensitized HCC cells to chemotherapy in vitro.^[^ ^49^ ^]^ Our study demonstrated that PK11195 inhibited the growth of xenograft tumors in nude mice by promoting ferroptosis (Figure 7A–D and Figure S8A,B, Supporting Information). It has been reported that ferroptosis inducers enhance the efficacy of anti‐PD‐1/PD‐L1 immunotherapy.^[^ ^13^ ^]^ Here, the combination of PK11195 with anti‐PD‐1 antibody exhibited synergistic antitumor effects and prolonged survival in an orthotopic mouse model (Figure 7E–H). Meanwhile, PK11195 had a favorable safety profile, which provided a basis for its potential clinical applications (Figure 7J and Figure S8C, Supporting Information). The tumor microenvironment (TME) is a complex system composed of tumor cells, immune cells, stromal cells, and cytokines. The immune‐suppressive TME is a critical hurdle for successful cancer immunotherapy, and remodeling the TME has become the key factor in immunotherapy. Therefore, in addition to T cell activation, the regulatory effect of PK11195 on other immune cells deserves further exploration. Our results have important implications for the development of combination strategies of ferroptosis inducers with ICBs in HCC.

In conclusion, in the present study we demonstrate that mitochondrial TSPO is highly expressed in HCC and correlated with poor prognosis. In addition, upregulated TSPO inhibits ferroptosis in HCC and antitumor immunity mediated by CD8^+^ T cells. Notably, the combination of TSPO inhibitors and ICBs provides an innovative therapeutic option for HCC. Therefore, targeting mitochondrial TSPO may be a promising new strategy for HCC treatment.

## 4 Experimental Section

### Ethics Statement, Patients, and Specimens

Human HCC tumor tissues and paired adjacent nontumor tissues were obtained from the First Affiliated Hospital with Zhejiang University School of Medicine (Zhejiang, China). This study was conducted with approval from the Research Ethics Committee of the First Affiliated Hospital with Zhejiang University School of Medicine (IIT20220776A). All samples were collected with the informed consent of the patients according to the guidelines of the 1975 Declaration of Helsinki.

### Ethics Statement and Animal Studies

All animal studies and experiments were approved by the Animal Research Committee of the First Affiliated Hospital with Zhejiang University School of Medicine (2022‐1528). BALB/c and C57BL/6 mice (male, SPF grade, 4–6 weeks old) were purchased from Hangzhou Medical College (Hangzhou, Zhejiang).

For subcutaneous tumor xenograft models, 5 × 10^6^ HCC cells (HCCLM3, MHCC97H, Huh7, Hep3B) suspended in 0.2 mL PBS were subcutaneously injected into the right flank of nude mice. The tumor size was measured and recorded every 7 days using calipers and the tumor volume was calculated according to the formula = (length × width^2^)/2. For lung metastasis models, similarly, 2 × 10^6^ HCC cells suspended in 0.1 mL PBS were injected into the tail vein of nude mice. For orthotopic tumor models, 3 × 10^5^ HCC cells (Hepa1‐6) suspended in 0.025 mL Matrigel were injected under the left liver capsule of anesthetized C57BL/6 mice. After injection 2–3 weeks, mice were sacrificed and livers were collected for histological and flow cytometric analyses. Combination therapy with PK11195 (MCE, China) and anti‐PD‐1 (BioXcell, USA) was accomplished using an orthotopic tumor model of C57BL/6 mice. On day 3 after Hepa1‐6 cells injection, the mice were randomly divided into four groups (*n* = 6) and treated intraperitoneally (i.p.) with PK11195 (20 mg kg^−1^, daily) or/and anti‐PD‐1 (100 µg/mouse, 3×/week) or rat IgG (control; BioXcell, USA). The luminescence images were taken using the IVIS Imaging System (Caliper Life Sciences, USA).

### Statistical Analysis

Data were presented as mean ± standard deviation (SD). The comparison of significances was evaluated by a two‐sided Student's *t* test for two groups and a one‐way ANOVA test for three or more groups. Correlation analyses were analyzed using a Pearson correlation test. The differences in survival were calculated by Kaplan–Meier analysis and the log‐rank test. Statistical analysis was carried out using SPSS (V23, IBM Corp, USA) and GraphPad Prism software (V9.0.2, GraphPad Inc., USA). A *p* value < 0.05 was considered statistically significant.

## Conflict of Interest

The authors declare no conflict of interest.

## Author Contributions

D.G.Z., D.M., and J.H.L. contributed equally to this work. D.G.Z., D.M., and J.H.L. performed the experiments, analyzed data, and wrote the manuscript. B.D. and R.L.T. performed animal experiments and analyzed data. Y.F.J. performed cell experiments and analyzed data. R.S. provided technical support. J.R.C. and B.Y. provided tissue specimens and clinical information. S.S.Z., D.Y.C., and J.W. supervised the study.

## Supporting information

Supporting Information

## Data Availability

The data that support the findings of this study are available in the supplementary material of this article.
