# Supplementary material for: Mitochondrial TSPO Promotes Hepatocellular Carcinoma Progression through Ferroptosis Inhibition and Immune Evasion
Source: Adv Sci (Weinh). 2023 Mar 30;10(15):2206669. doi: 10.1002/advs.202206669 (PMC10214260; doi:10.1002/advs.202206669)
Supplement: Supplementary file 1 — Supporting Information [file ADVS-10-2206669-s001.pdf]

Mitochondrial TSPO promotes hepatocellular carcinoma progression through ferroptosis inhibition and immune evasion

Deguo Zhang[1#], Da Man[1#], Jiahua Lu[1#], Yifan Jiang[1,2], Bo Ding[1,3], Rong Su[2,3,4], Rongliang Tong[1], Junru Chen[1], Beng Yang[1], Shusen Zheng[1,2,3,4*], Diyu Chen[1,4*], Jian Wu[1,2,3,4*]

Supplementary Methods

Immunohistochemistry: Tissue samples were fixed in 10% neutral buffered formalin, embedded in paraffin, and sectioned to 4 μm slides. After dewaxing and rehydration, tissue antigens were retrieved by heating the slides using citrate buffer or EDTA solution. Samples were incubated with primary antibodies at 4°C overnight and with appropriate biotinylated secondary antibodies. Then the sections were analyzed using a diaminobenzidine (DAB) chromogen kit and counterstained with hematoxylin. The intensity of protein expression was calculated from both the percentage (0: 0%, 1: 1-25%, 2: 26-75%, 3: >75%) and intensity (0: negative, 1: low, 2: medium, 3: high) of positively stained cells. The final histoscore was calculated by multiplying the percentage score and intensity score.

Quantitative Real-Time PCR: Total RNA was extracted from cells or tumor tissues using RNA isolater Total RNA Extraction Reagent (Vazyme, China), and reverse transcription reactions were performed with MonScript™ RTIII All-in-One Mix with dsDNase kit (Monad, China) according to the manufacturer's instructions. Quantitative real-time PCR was performed using ChamQ Universal SYBR qPCR master mix (Vazyme, China) in a QuantStudio 5 Real-Time PCR System (Thermo Fisher, USA). The expression of target genes was calculated using the $2^{-\Delta\Delta Ct}$ method and normalized to the expression of GAPDH. The sequences of primers were listed in **Supplementary Table 3**.

Western blot: The samples were lysed in RIPA Buffer (Servicebio, China) supplemented with protease and phosphorylation inhibitor cocktail (Selleck, USA). Equal amounts of protein were separated by SDS-PAGE and transferred to PVDF membranes (Millipore, USA). After blocking with

5% nonfat milk, the membranes were incubated overnight at 4 °C with primary antibodies and then incubated with HRP-conjugated secondary antibodies (1:5000; GenScript, China) for 1 h. The blots were detected with an ECL detection reagent (Fudebio, China) and measured using the Image J software. The antibodies were listed in **Supplementary Table 4**.

Cell lines and culture conditions: Human HCC cell lines HCCLM3, MHCC97H, Huh7, Hep3B, PLC/PRF/5, and Li-7 were purchased from the Cell bank of Chinese Academy of Science (Shanghai, China). Mouse HCC cell line Hepa1-6 was purchased from the American Type Culture Collection (Manassas, USA). Cells were cultured in DMEM supplemented with 10% fetal bovine serum (FBS; BioInd, Israel)), 100 IU/mL penicillin, and 100 IU/mL streptomycin and were kept in a humidified 5% CO_2 incubator at 37°C.

Cell transfection: For transient silencing, small interfering RNAs (siRNA) and negative control (NC) siRNAs were designed and chemically synthesized by SUNYA Biotechnology (Zhejiang, China). For stable silencing, small hairpin RNAs (shRNA) lentivirus were produced by transfection of HEK293T cells with recombinant lentivirus vectors and lentivirus packaging plasmids. All the plasmids used in this study were synthesized and purchased from RiboBio (Guangzhou, China). Samples were transfected using jetPRIME (Polyplus, France) transfection reagent in accordance with the manufacturer's protocol. The sequences of siRNAs and shRNAs used for transfection are shown in **Supplementary Table 5**.

CCK-8 and colony formation assay: Cell proliferation was assessed by CCK-8 assay (MCE, USA).

Cells (2×10^3 cells/well) were plated in 96 well plates with 200 μl of culture medium and cultured for the indicated time. With supernatant removed, CCK-8 reagent was added to each well and incubated for 1 h at 37 °C. The absorbance at 450 nm was detected using a microplate reader (Bio-Rad, USA).

For colony formation assay, cells (1×10^3 cells/well) were seeded into 6 well plates and incubated for 2-3 weeks. Then, the colonies on the plates were fixed with 4% polyoxymethylene and stained with 0.1% crystal violet.

Transwell and Wound healing assays: For migration and invasion assays, cells (5×10^4) suspended in serum-free medium were seeded into each upper chamber with or without pre-coated Matrigel (Corning, USA), while medium containing 10% FBS was added to the lower chamber. After incubation for 48 h, the migratory or invasive cells in the lower chambers were fixed with 4% paraformaldehyde and stained with crystal violet.

For wound healing assay, cells (5×10^4) were seeded into both sides of Culture-Inserts® (Ibidi, Germany) to make a 500 μm gap. After incubation for 24 h, inserts were removed to allow cell migration for the indicated period of time.

Immunofluorescence: Cells were fixed in 4% paraformaldehyde (PFA) for 15 min and permeabilized with 0.2% Triton X-100 for 30 min. After being blocked with 5% bovine serum albumin (BSA) for 1 h, the cells were incubated with primary antibodies overnight at 4 °C. Then the cells were stained with secondary antibodies (Proteintech, China) for 1 h at room temperature, and with DAPI (Servicebio, China) for 5 min. Images were taken with a Leica TCS SP8 confocal fluorescence

microscope system (Leica, Germany).

Co-immunoprecipitation (Co-IP): Cells were lysed in IP/Western lysis buffer (Beyotime, China) containing a proteinase and phosphatase inhibitor cocktail (Selleck, USA). The cell lysates were incubated with antibodies in a rotating incubator overnight at 4 °C, following the co-incubation with protein A/G magnetic beads (Bimake, China) at 4 °C for 6 h. Beads were washed with PBST and boiled in 1× SDS-loading buffer (GenScript, China) and the binding proteins were analyzed by LC-MS/MS or Western blot analysis.

GST pull-down: The GST-tagged fusion protein (GST-TSPO) and His-tagged fusion protein (His-P62) were expressed in BL21 (DE3) Escherichia coli induced by isopropylthiogalactoside (IPTG, Solarbio, China). The proteins were purified using the BeyoGold™ GST-tag Purification Resin and BeyoGold™ His-tag Purification Resin (Beyotime, China) following the manufacturer's instructions. For GST pull-down assay, purified GST-TSPO protein was incubated with purified His-P62 protein and glutathione-Sepharose 4B beads at 4 °C for 6 h by rotation. After washing with binding buffer (Sango biotech, China), proteins bound to the beads were eluted by boiling with 1×SDS-loading buffer and then analyzed by Western blot.

Transmission electron microscopy (TEM): Cells were collected centrifugally and fixed with the 2.5% glutaraldehyde solution at 4 °C overnight. After fixation using osmium acid and uranium acetate, samples were dehydrated by a graded series of ethanol and acetone, followed by embedment in resin mixture. Ultrathin sections were cut and stained and observed in Spirit 120 kV TEM (Tecnai, USA).

Determination of intracellular ROS, iron, and lipid peroxidation: Cells (1×10^5 cells/well) were seeded into 12 well plates and treated for the indicated times. For detection of ROS, iron, and lipid peroxidation, cells were incubated with DCFH-DA (Beyotime, China), FerroOrange (Dojindo, Japan), Mito-FerroGreen (Dojindo, Japan), and C11-BODIPY 581/591 (Abclonal, China) in a serum-free medium at 37 °C in the dark. Subsequently, cells were washed three times with PBS or serum-free medium and digested into cell suspension analyzed by flow cytometry (BD FACSCanto II, USA).

GSH /GSSG and cholesterol assay: After being washed with PBS, equal numbers of cells were collected using a Vi-Cell cell counter (Beckman Coulter, USA). The intracellular level of GSH (reduced glutathione)/GSSG (oxidized glutathione disulfide) and cholesterol was measured using GSH/GSSG Assay Kit (Beyotime, China) and Free Cholesterol Content Assay Kit (Applygen, China), according to the manufacturer's protocols.

Luciferase reporter Assay: Human PD-L1 promoter fragments were synthesized and inserted into the pGL3-Promoter vector. Cells were seeded on 24-well plates and transfected with truncated or mutated PD-L1 promoter luciferase reporter plasmid, pRL-TK (Renilla luciferase) reporter plasmid, and Flag-Nrf2 or negative control. The luciferase reporter assays were carried out on cell lysates with the Dual Luciferase Reporter Assay Kit (Vazyme, China) according to the manufacturer's instructions. The relative firefly luciferase activity was normalized to co-transfected renilla luciferase activity.

Chromatin immunoprecipitation (ChIP) Assay: ChIP assay was conducted with the SimpleChIP® Enzymatic Chromatin IP Kit (Magnetic Beads, CST, USA) according to the manufacturer's protocol. The enrichment of DNA sequences was analyzed by quantitative real-time PCR, and the primers were listed in **Supplementary Table 3**.

Flow cytometry: Cells from the mouse tumors were obtained by grinding the tissue through 70 um filters. After incubated with Fc block, cells were stained with surface or intracellular primary antibodies (Biolegend, USA) according to the manufacturer's protocols. Stained cells were acquired on a Flow Cytometer (BD FACSCanto II or BD LSRFortessa, USA) and the results were analyzed by FlowJo software. The antibodies used for flow cytometry are listed in **Supplementary Table 4**.

Table S1. Correlations between TSPO expression and clinicopathologic features of HCC patients

Characteristics		Tumor TSPO expression		p value
		Low (n=37)	High (n=43)	
Age (y)	≤50	17	15	0.314
	>50	20	28	
Gender	Male	34	39	0.851
	Female	3	4	
HBsAg	Negative	5	12	0.117
	Positive	32	31	
HBcAb	Negative	3	4	0.851
	Positive	34	39	
HCVAb	Negative	36	40	0.382
	Positive	1	3	
ALT (U/L)	≤50	23	29	0.622
	>50	14	14	
ALB (g/L)	≤35	7	16	0.072
	>35	30	27	
AFP (ng/ml)	≤400	22	31	0.233
	>400	15	12	
GGT (U/L)	≤45	16	16	0.583
	>45	21	27	
TB (umol/L)	≤17.1	27	30	0.752
	>17.1	10	13	
Tumor encapsulation	Complete	14	21	0.323
	None	23	22	
Tumor differentiation	I-II	23	15	**0.015**
	III-IV	14	28	
Tumor size (cm)	≤5	25	30	0.832
	>5	12	13	
Tumor number	Single	33	36	0.479
	Multiple	4	7	
Liver cirrhosis	No	3	6	0.409
	Yes	34	37	
TNM stage	I-II	30	25	**0.027**
	III-IV	7	18	

P value was calculated by chi-square test. *P* value < 0.05 was considered statistically significant.

Abbreviations: HBsAg, hepatitis B surface antigen; HbcAb, hepatitis B core antibody; HCVAb, hepatitis C virus antibody; ALT, alanine aminotransferase; ALB, albumin; AFP, alpha-fetoprotein.

Table S2. Univariate and multivariate analysis of factors associated with overall and recurrence free survival

Variables	OS	RFS

	Univariate	Multivariate		Univariate	Multivariate	
	P	*P*	HR (95%CI)	*P*	*P*	HR (95%CI)
Age(＞50 years vs. ≤ 50 years)	0.669	NA		0.924	NA	
Gender (female vs. male)	0.504	NA		0.985	NA	
HBsAg (positive vs. negative)	0.818	NA		0.894	NA	
ALT (＞50 vs. ≤ 50)	0.877	NA		0.370	NA	
ALB (＞35 vs. ≤ 35)	0.571	NA		0.123	NA	
AFP (＞400 vs. ≤ 400)	0.735	NA		0.376	NA	
Tumor encapsulation (none vs. complete)	0.058	**0.046**	**1.942 (1.011-3.729)**	0.368	NA	
Tumor differentiation (III-IV vs. I-II)	0.102	NA		**0.004**	NS	
Tumor size (＞5 vs. ≤ 5)	0.082	NS		**0.040**	**0.023**	**2.127 (1.112-4.070)**
Tumor number (multi vs. single)	0.062	NS		**0.014**	**0.014**	**2.600 (1.218-5.550)**
Liver cirrhosis (Yes vs. No)	0.428	NA		0.345	NA	
TNM stage (III-IV vs. I-II)	0.083	NS		0.495	NA	
TSPO (high vs. low)	**0.027**	**0.035**	**1.445 (1.027-2.035)**	**0.007**	**0.038**	**1.410 (1.020-1.948)**

P value was calculated using Cox proportional hazards regression. *P* value < 0.05 was regarded as statistically significant.

Abbreviations: HBsAg, hepatitis B surface antigen; ALT, alanine aminotransferase; ALB, albumin; AFP, alpha-fetoprotein; NA, not adopted;

Table S3. Primers for quantitative real-time PCR and ChIP-PCR analysis

Gene	Primers
hGAPDH-F	GGAGCGAGATCCCTCCAAAAT
hGAPDH-R	GGCTGTTGTCATACTTCTCATGG
hTSPO-F	TTCACAGAGAAGGCTGTGGTTC
hTSPO-R	GCCATACGCAGTAGTTGAGTGT
hP62-F	TGTGTAGCGTCTGCGAGGGAAA
hP62-R	AGTGTCCGTGTTTCACCTTCCG
hNrf2-F	CACATCCAGTCAGAAACCAGTGG
hNrf2-R	GGAATGTCTGCGCCAAAAGCTG
hPD-L1-F	TGCCGACTACAAGCGAATTACTG
hPD-L1-R	CTGCTTGTCCAGATGACTTCGG
hGPX4-F	ACAAGAACGGCTGCGTGGTGAA
hGPX4-R	GCCACACACTTGTGGAGCTAGA
hHO-1-F	CCAGGCAGAGAATGCTGAGTTC
hHO-1-R	AAGACTGGGCTCTCCTTGTTGC
hNQO1-F	CCTGCCATTCTGAAAGGCTGGT
hNQO1-R	GTGGTGATGGAAAGCACTGCCT
hGCLC-F	GGAAGTGGATGTGGACACCAGA
hGCLC-R	GCTTGTAGTCAGGATGGTTTGCG
hGCLM-F	TTGGAGTTGCACAGCTGGATTC
hGCLM-R	TGGTTTTACCTGTGCCCACTG
hPPAR-γ-F	AGCCTGCGAAAGCCTTTTGGTG
hPPAR-γ-R	GGCTTCACATTCAGCAAACCTGG
mGAPDH-F	CATCACTGCCACCCAGAAGACTG
mGAPDH-R	ATGCCAGTGAGCTTCCCGTTCAG
mTSPO-F	GAGCCTACTTTGTACGTGGCGA
mTSPO-R	GCTCTTTCCAGACTATGTAGGAG
mPD-L1-F	GCTCCAAAGGACTTGTACGTG
mPD-L1-R	TGATCTGAAGGGCAGCATTTC
PD-L1-WT1-F	GGGCTTTCTTAACCCTCACC
PD-L1-WT1-R	GACCCATATGGCTTTGGTTTT
PD-L1-WT2-F	ATGGGAAAATGAATGGCTGA
PD-L1-WT2-R	CTCCTAGATGGCCTGGATGA
PD-L1-WT3-F	TCATCCAGGCCATCTAGGAG
PD-L1-WT3-R	CTATCTGCAATGCCCTCTGA

Table S4. Antibodies used in this study

Antibodies	Company	Cat no.	Clone no.
GAPDH	Proteintech	10494-1-AP	
TSPO	Abcam	ab109497	EPR5384
P62	Abcam	ab109012	EPR4844
P62	Proteintech	66184-1-Ig	1H5C1
P62	Abcam	ab56416	2C11
KEAP1	Abcam	ab119403	1B4
KEAP1	Proteintech	10503-2-AP	
Nrf2	Abcam	ab62352	EP1808Y
Nrf2	CST	12721	D1Z9C
Nrf2	Proteintech	16396-1-AP	
E-Cadherin	CST	3195T	24E10
N-Cadherin	CST	13116T	D4R1H
Vimentin	CST	5741T	D21H3
Snail	CST	3879T	C15D3
HO-1	Abcam	ab52947	EP1391Y
NQO1	Abcam	ab80588	EPR3309
GCLC	Abcam	ab190685	EP13475
GCLM	Abcam	ab126704	EPR6667
GPX4	Abcam	ab125066	EPNCIR144
PPAR-γ	Abcam	ab178860	EPR18516
CD4	Abcam	ab183685	EPR19514
CD8	CST	98941	D4W2Z
PD-L1	CST	60475	D4H1Z
PD-L1	CST	13684	E1L3N
Ki67	Abcam	ab15580	
PCNA	Abcam	ab29	PC10
PD-1	Bio X Cell	BE0146	RMP1-14
IgG2a isotype	Bio X Cell	BE0089	2A3
PD-L1	Biolegend	329706	29E.2A3
IgG2b isotype	Biolegend	400314	MPC-11
PD-L1	Biolegend	124311	10F.9G2
IgG2b isotype	Biolegend	400611	RTK4530
CD45, PE	Biolegend	147712	I3/2.3
CD3, APC	Biolegend	100236	17A2
CD3, BV421	Biolegend	100228	17A2
CD4, PE/Cyanine 7	Biolegend	100422	GK1.5
CD4, BV605	Biolegend	100451	GK1.5
CD8a, FITC	Biolegend	100706	53-6.7
CD8a, AF700	Biolegend	100729	53-6.7
Granzyme B, PE/Cyanine 7	Biolegend	372214	QA16A02
TNF-α, PE	Biolegend	506306	MP6-XT22

Table S5. Sequences of shRNAs and siRNAs in this study

Name	Sequences
hTSPO-shRNA-1	CCACACTCAACTACTGCGTAT
hTSPO-shRNA-2	CGGCTCCTACCTGGTCTGGAA
mTSPO-shRNA	GGUAUGCUAGCUUGCAGAA
hNrf2-shRNA-1	GCAGTTCAATGAAGCTCAA
hNrf2-shRNA-2	GAGTTACAGTGTCTTAATA
hTSPO-siRNA-1	Sense 5′- CCACACUCAACUACUGCGUAUTT-3′
hTSPO-siRNA-1	Anti-sense 5′- AUACGCAGUAGUUGAGUGUGGTT-3′
hTSPO-siRNA-2	Sense 5′- CGGCUCCUACCUGGUCUGGAATT-3′
hTSPO-siRNA-2	Anti-sense 5′- UUCCAGACCAGGUAGGAGCCGTT-3′

Supplementary Figures and Figure legends

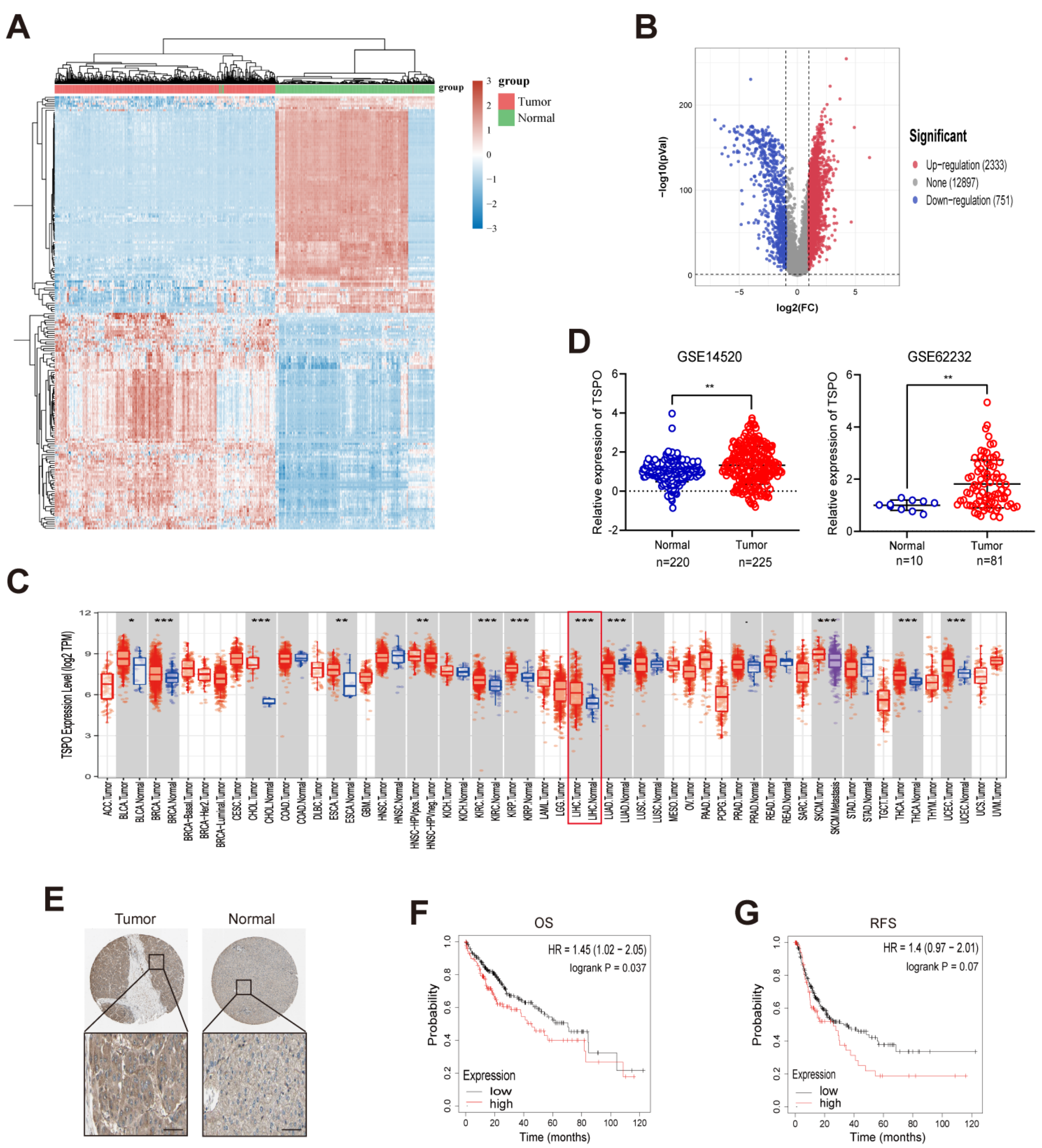

SFig. 1 TSPO is upregulated and associated with a poor prognosis in HCC

(A, B) Heatmaps and volcano plots represent differentially expressed genes between tumor and normal tissues in the TCGA and GTEx databases ($p<0.05$; |LogFC| >1). (C) TSPO mRNA expression in different tumor tissues based on the TIMER database. (D) TSPO mRNA expression in normal

liver tissues and HCC tissues from the GEO database. (E) Representative IHC images of TSPO expression from the HPA database. (F, G) Survival analyses of OS and RFS in different TSPO expression groups from the KM Plotter database.

*$P<0.05$, **$P<0.01$, ***$P<0.001$. LIHC, liver hepatocellular carcinoma; OS, overall survival; RFS, recurrence free survival.

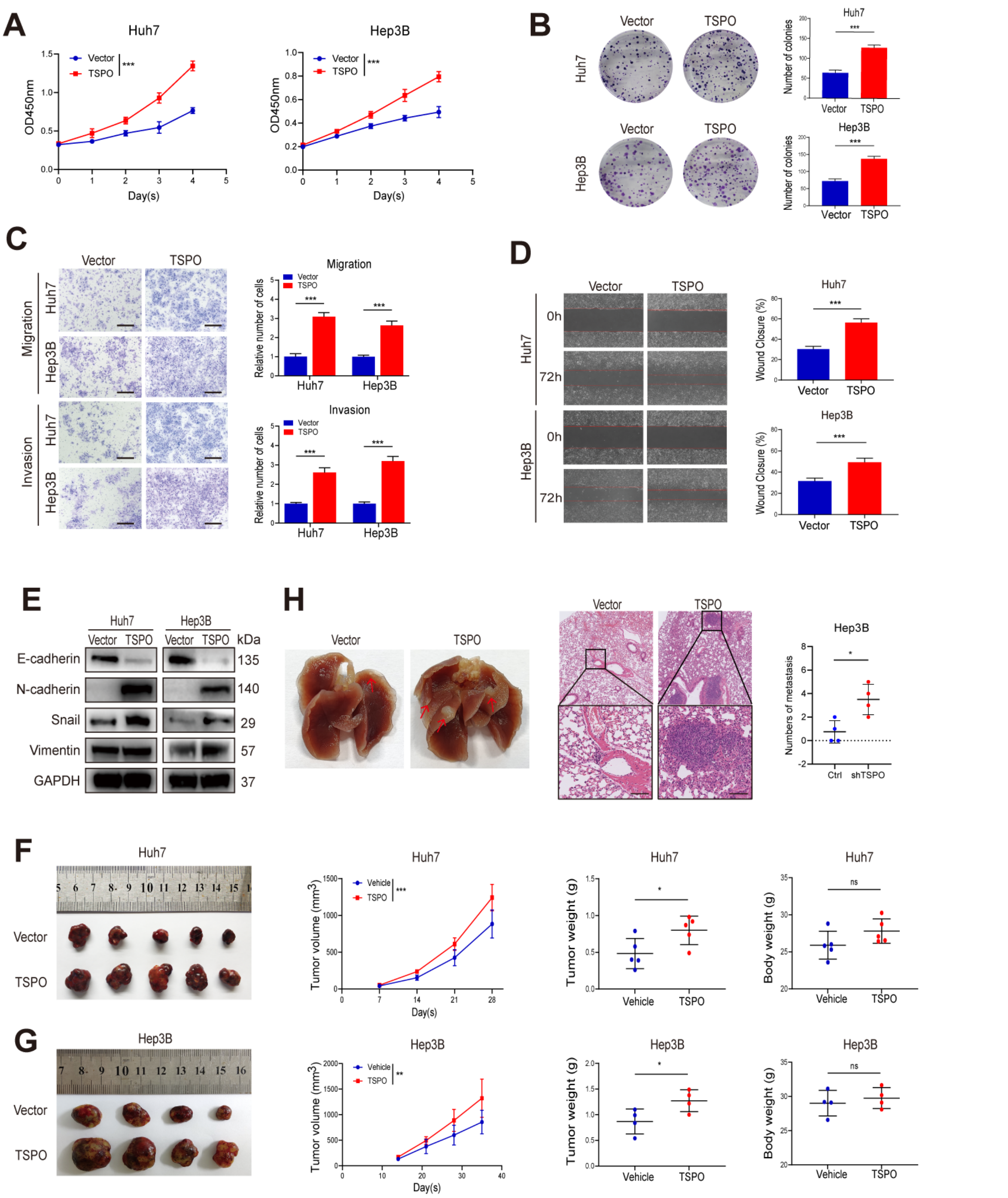

SFig. 2 Overexpression of TSPO promotes HCC cell proliferation, invasion and metastasis in vitro and in vivo

(A, B) The proliferation of Huh7 and Hep3B cells was detected by CCK-8 and colony formation assays. (C, D) The migratory and invasive capabilities of Huh7 and Hep3B cells were evaluated

using wound healing and transwell assays (scale bar, 200 μm). (E) The protein levels of EMT markers in Huh7 and Hep3B cells were detected by Western blot. (F, G) Images of xenograft tumors in nude mice and statistical analyses of tumor volumes, tumor weights and body weights in the different groups. (H) Representative images and quantification of metastatic lung nodules in nude mice (red arrows marked, , n=4).

**P*<0.05, ***P*<0.01, ****P*<0.001. n.s.: not significant. The data are expressed as the mean±SD of three independent experiments.

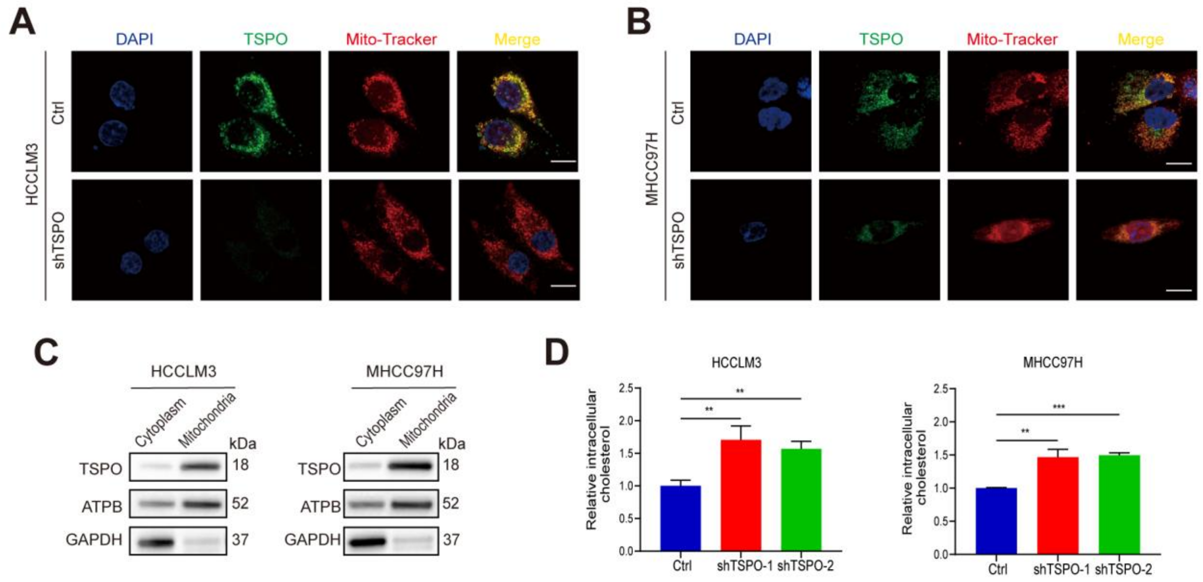

SFig. 3 TSPO inhibits ferroptosis in HCC cells

(A, B) Immunofluorescence assay confirmed the colocalization of TSPO (green) and mitochondria (labeled with MitoTracker red; scale bar, 20 μm). (C) Western blot analysis of TSPO protein expression in mitochondria and cytoplasm. (D) The intracellular cholesterol level was measured enzymatically using a commercial kit.

***P*<0.01, ****P*<0.001. The data are expressed as the mean±SD of three independent experiments.

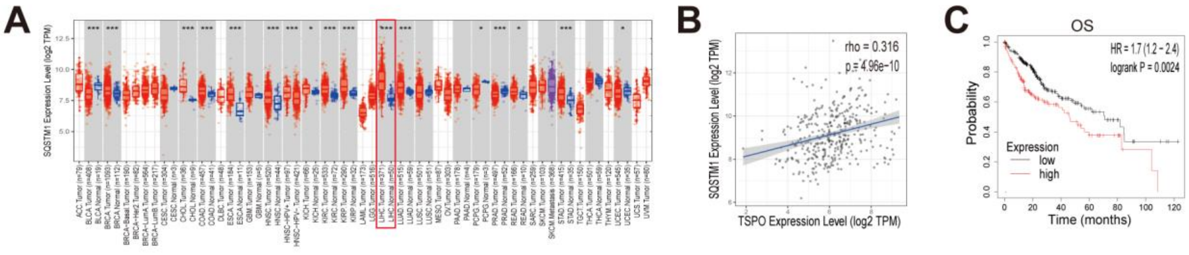

SFig.4 TSPO mediates autophagy inhibition and P62 accumulation via interaction with P62

(A) P62 mRNA expression in different tumor tissues based on the TIMER database. (B) Correlation analysis of TSPO and P62 was performed from the TIMER database. (C) Kaplan-Meier curve showing that higher P62 expression was associated with a poor OS from the KM Plotter database.

**P*<0.05, ****P*<0.001.

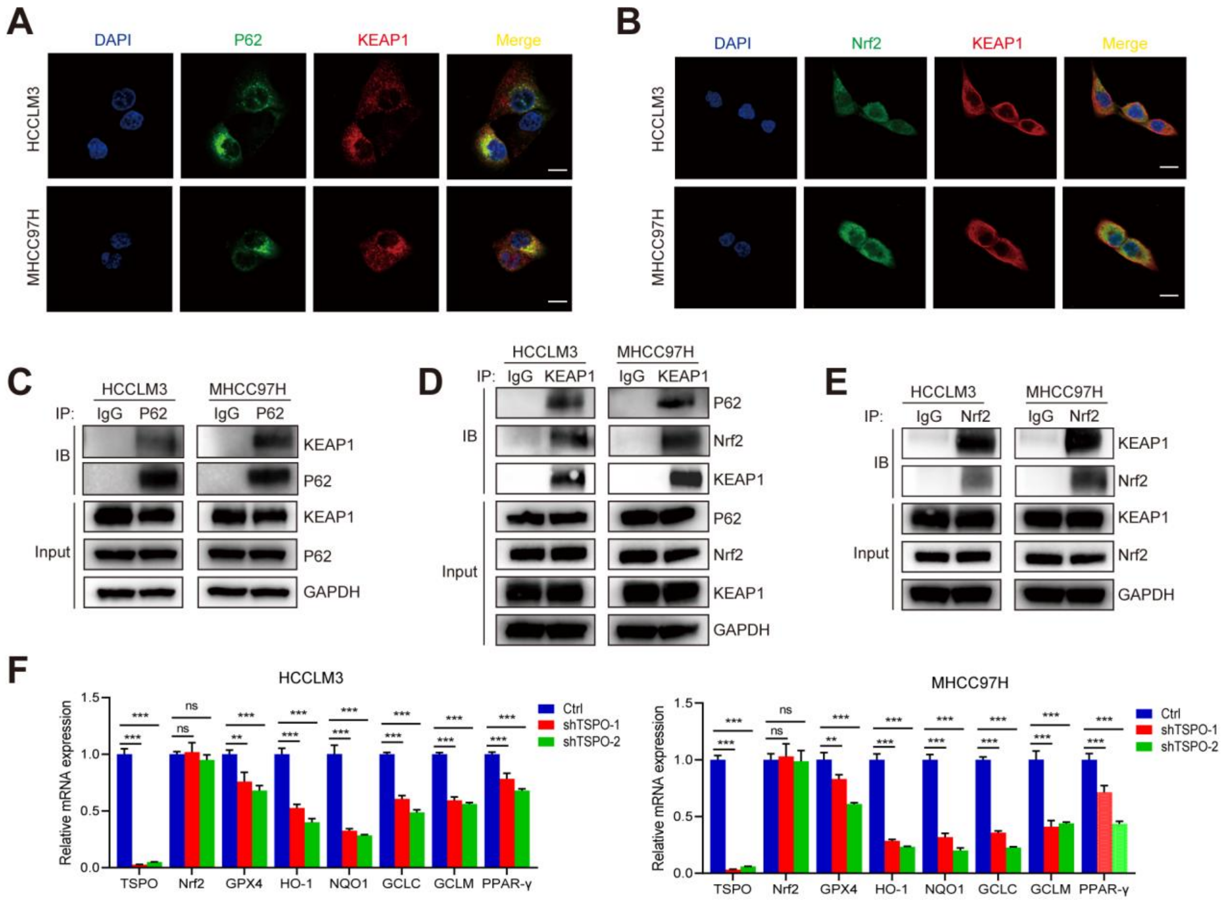

SFig. 5 TSPO inhibits ferroptosis through P62/KEAP1/Nrf2 antioxidant pathway

(A, B) Immunofluorescence assay showed the colocalization of P62, KEAP1 and Nrf2 in HCCLM3 and MHCC97H cells (scale bar, 20 μm). (C, D, E) Co-IP assay showed the protein interaction between P62, KEAP1, and Nrf2 in HCCLM3 and MHCC97H cells. (F) The mRNA levels of downstream antioxidant genes regulated by Nrf2 in HCCLM3 and MHCC97H cells were detected by qRT-PCR.

*P<0.05, **P<0.01, ***P<0.001. The data are expressed as the mean±SD of three independent experiments. Co-IP, co-immunoprecipitation.

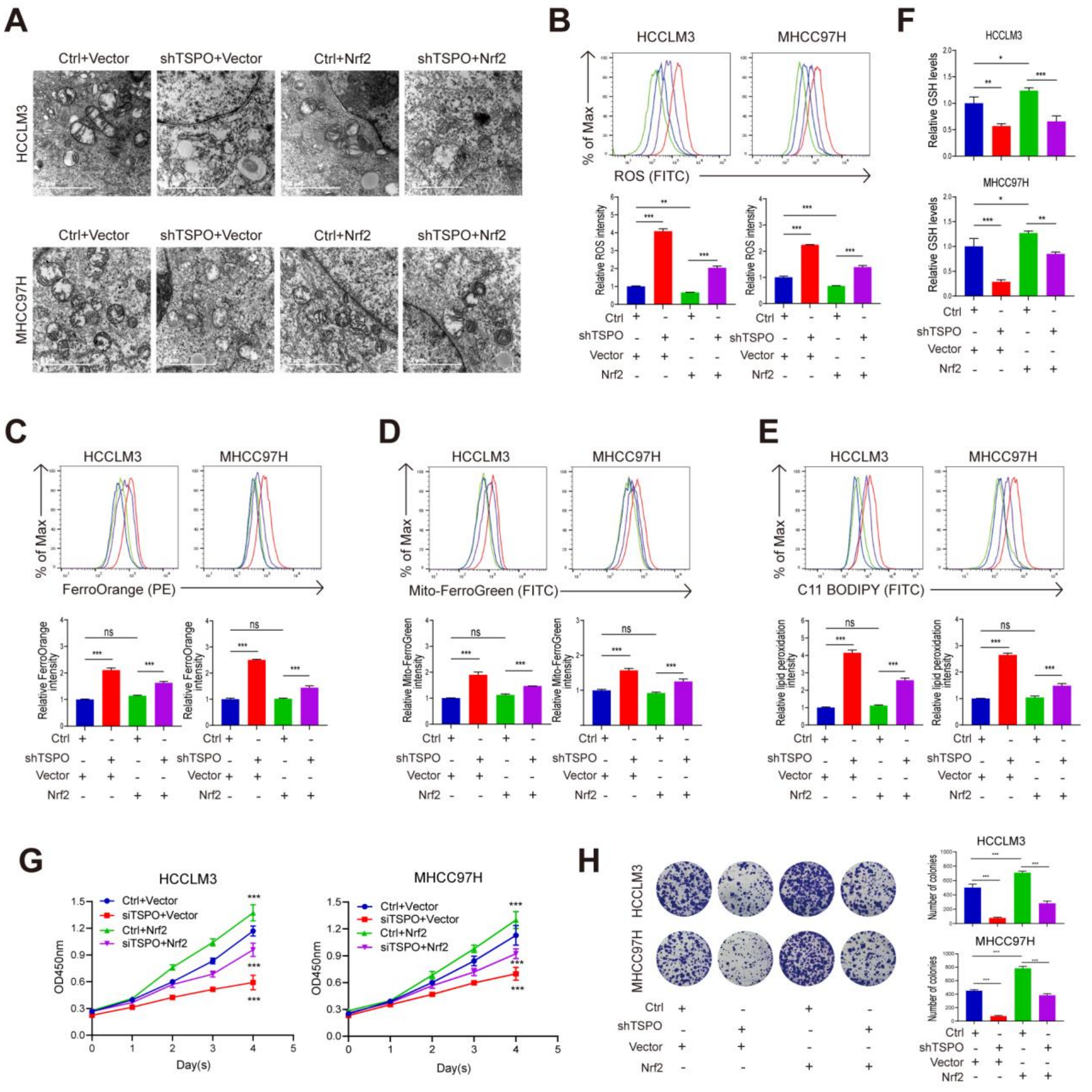

SFig. 6 Nrf2 overexpression reverses ferroptosis and phenotype changes induced by TSPO knockdown

(A) The morphology of mitochondria in HCCLM3 and MHCC97H cells was observed by TEM. (B) The intracellular ROS of HCCLM3 and MHCC97H cells was detected by flow cytometry using DCFH-DA. (C, D) The intracellular and intramitochondrial $Fe2^{+}$ of HCCLM3 and MHCC97H cells were detected by flow cytometry using FerroOrange and Mito-FerroGreen, respectively. (E) The

accumulation of lipid ROS of HCCLM3 and MHCC97H cells was detected by flow cytometry using C11-BODIPY 581/591. (F) The intracellular GSH of HCCLM3 and MHCC97H cells was detected using a GSSG/GSH quantification kit. (G, H) The proliferation of HCCLM3 and MHCC97H cells was detected by CCK-8 and colony formation assays.

*P<0.05, **P<0.01, ***P<0.001. The data are expressed as the mean±SD of three independent experiments.

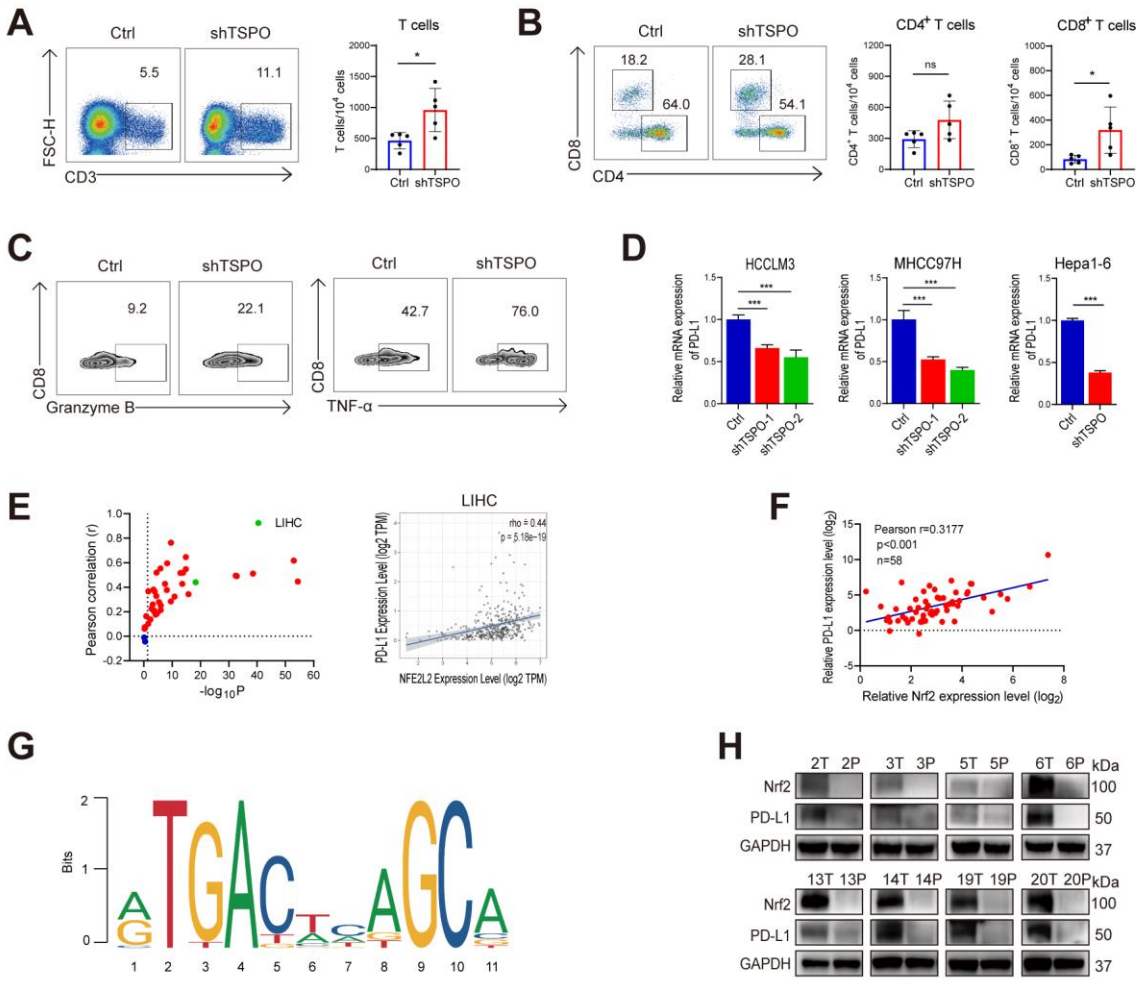

SFig. 7 TSPO promotes tumor immune escape by upregulating PD-L1 expression through Nrf2-mediated transcription

(A, B, C) Representative images and statistical analysis of tumor-infiltrating lymphocytes were analyzed by flow cytometric. (D) The mRNA levels of PD-L1 in HCCLM3, MHCC97H and Hepa1-6 cells were detected by qRT-PCR. (E) Correlation analysis of Nrf2 and PD-L1 was performed in various tumors from the TIMER database. (F) Correlation analysis of Nrf2 and PD-L1 was performed in HCC clinical specimens by qRT-PCR. (G) The eleven-base motifs of Nrf2 were obtained from the Jaspar database. (H) Correlation analysis of Nrf2 and PD-L1 was performed in HCC clinical specimens by Western blot.

***$P<0.001$. The data are expressed as the mean±SD of three independent experiments.

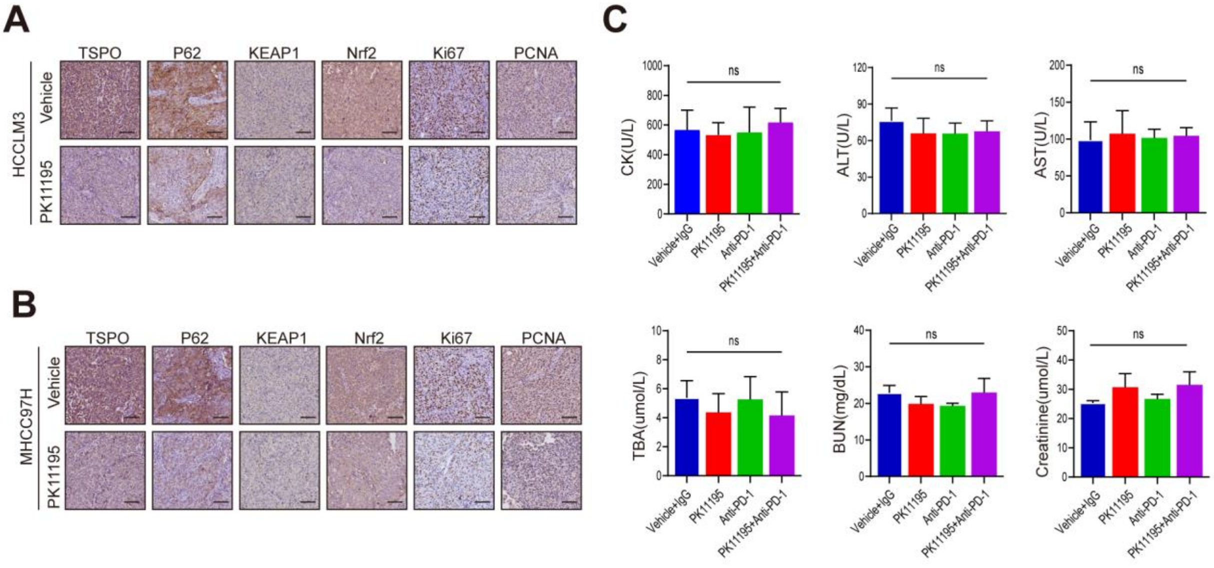

SFig. 8 TSPO inhibitor promotes ferroptosis and improves the anti-PD-1 efficacy in a mouse model

(A, B) Representative images of IHC staining with indicated antibodies in subcutaneous xenograft tumor tissues. (C) Serum levels of the heart, liver and kidney function indicators in C57/BL6 mice after PK11195 and anti-PD-1 antibody treatment.

n.s.: not significant.
